# Congenitally Corrected Transposition of Great Arteries with Severe Rhythm Disturbances

**Published:** 2010-04-01

**Authors:** Mehmet Kayrak, Zeynettin Kaya, Enes Elvin Gul, Mehmet S Ulgen, Mehmet Yazici, Serter Gumus, Yahya Paksoy

**Affiliations:** 1 Selcuk University, Meram Faculty of Medicine, Department of Cardiology, Konya / Turkey; 2 Selcuk University, Meram Faculty of Medicine, Department of Radiology, Konya / Turkey

**Keywords:** congenitally corrected transposition of great arteries, arrhythmias

## Abstract

Within recent years, much scientific attention has been devoted to adults with congenital heart disease (CHD) and probable complications. Congenitally corrected transposition of the great arteries (CCTGA) is a rare, complex form of congenital heart defects. CCTGA is characterized by atrioventricular (AV) and ventriculoarterial (VA) discordance and, hence, by a physiologically normal direction of blood flow. The development of complete AV block and global ventricular dysfunction has been identified as the cause of cardiac death. Although the development of arrhythmias represents a major cause of morbidity and mortality in patients with CHD, the account of all implantations of pacemakers and implantable cardioverter defibrillators (ICD) is less than one percent. This paper presents a case of CCTGA with severe rhythm disorders, discusses probable treatment options, and offers indications of ICD implantation in patients with CHD.

##  Introduction

CCTGA has wide spectrum of structural and clinical features. The clinical presentation and prognosis of patients with CCTGA vary depending on the severity of the associated cardiac anomalies, the development of systemic ventricular dysfunction, and the development of arrhythmias. The patients with CCTGA have a progressive risk of spontaneous complete AV block throughout life (2% per year) [1]. The incidence of sudden cardiac death (SCD) in CHDs is approximately 1:1000 patients per year, which is 25-100 times greater than in the general population [2]. Despite the relatively common occurrence of SCD, ventricular tachycardia (VT) has been rarely described in the natural history of CCTGA.

## Case Report

**Figure 1:**
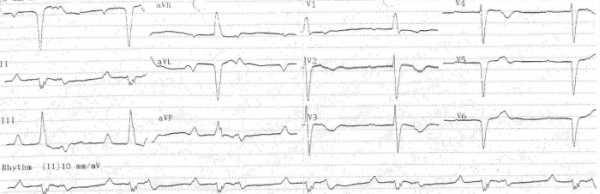
The patient was presented with an atrial rhythm with 2:1 AV block

**Figure 2:**
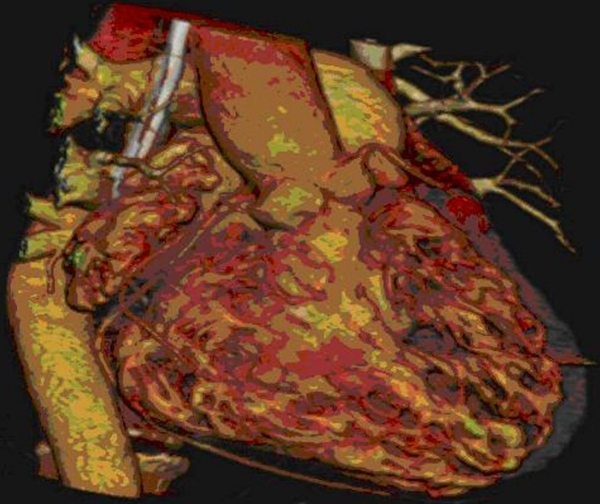
Cardiac MR showed aortic root arising from trabecular right ventricle, right and left coronary arteries originating from aortic root

A 56-year-old male was referred to the emergency department (ED) for fatigue and shortness of breath on exertion, for 3 - 4 days. The patient was bradycardic, and his blood pressure was 160/70 mmHg. Electrocardiography (ECG) showed an atrial rhythm with 2:1 AV block (Figure 1). The patient had no history of syncope. He was hospitalized and monitored in the Coronary Care Unit (CCU). His serial cardiac markers and laboratory data were normal, and there was no reversible cause to explain AV block. A chest X-ray showed slight cardiomegaly. A transthorasic echocardiographic evaluation detected an apically localized systemic AV valve, a dilated left atrium, a parallel arrangement of great arteries, significant systemic AV valve insufficiency, and ejection fraction (EF) of 32%. Multi-slice CT and MR angiography revealed AV and ventricular arterial discordance with persistent left superior vena cava and situs inversus abdominalis (Figure 2). Cardiac catheterization was performed. Selective coronary arteriography demonstrated a well-developed right coronary system.

**Figure 3:**
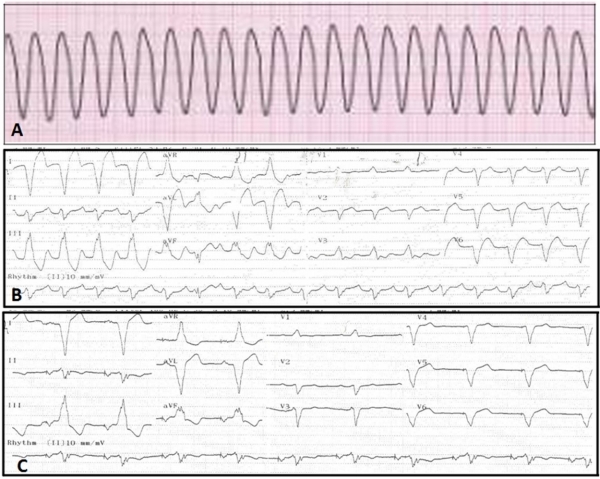
A: The patient suffered the first attack of ventricular tachicardia (VT) in the coronary care unite. VT was detected in the monitor. B: Atrial tachcardia with AV block was emerged and respiration was returned after cardiopulmonary resuscitation. C: ECG after the DDD-R ICD implantation

The patient experienced a sudden cardiac arrest during his follow-up in the CCU. The patient was monitored and intubated immediately. Pulseless ventricular tachycardia was detected (Figure 3A), direct electrical cardioversion was accomplished, and rhythm was restored. The ECG showed atrial tachycardia (Figure 3B) after the successful resuscitation. An electrophysiologic study was performed, but ventricular tachycardia was not induced again. The patient underwent dual-chamber ICD implantation with active fixation (Figure C). The patient was then discharged in clinically stable condition with spironolactone 25 mg, lisinopril 10mg, and bisoprolol 5mg treatment. There were no complaints during routine follow up at one month.

In a routine follow up in the sixth month, the patient was admitted with palpitation. Paroxysmal atrial fibrillation was detected by 24-hour continuous ambulatory ECG. The ICD was checked, and atrial tachycardias with variable conductions were detected. Amiadarone 600 mg and warfarin 5mg daily were started. We did not detect any recurrent attack of atrial fibrillation during the 6 months follow-up after the amiodorane therapy.

## Discussion

CCTGA, or synonym l-transposition, is a rare (less than 1% of all CHD) and complex heart defect. The characteristic feature of this CHD is AV and ventriculoarterial discordance. The great arteries are generally parallel to each other. The aorta is located closer to the anterior and more to the left than the pulmonary artery. The AV valves follow their respective ventricles. Because of the displacement of the AV node and the abnormal course of conduction tissue, there is an increased risk of spontaneous complete AV block. One study examined a series of patients with CCTGA, with complete AV block detected at 8% of diagnosis; the follow-up showed that 38% patients had been documented as experiencing atrial arrhytmias [3]. Associated cardiac defects are common; isolated CCTGA is an exception.

For a number of patients with CHD, life expectancy increases with the development of diagnostic and interventional techniques. SCD is still the leading cause of death in patients with CHD. CCTGA has the highest mortality among all CHD patients [4]. Despite the relatively common occurrence of SCD, VT has rarely been described in the natural history of CCTGA. Although the mechanism of VT in patients with CCTGA is not clear, it is likely related to triggered automaticity and reentry because of the progressive systemic ventricular dysfunction. There is a general acceptance of ICD implantation in patients with severe ventricular dysfunction for primary prevention. Patients with CHD, however, have not yet been shown to benefit from ICD placement for primary prevention. The Toronto study shows that sudden death is the most common mode of mortality in Tetralogy of Fallot, Ebstein’s anomaly, CCTGA, and congenital aortic valve anomaly, suggesting that these groups may benefit even more from primary prevention ICD implant [5]. The main indications for ICD implantation, according to the existing guidelines for patients with CHD, are resuscitated cardiac arrest, sustained VT in the absence of a reversible cause, and syncope with inducible sustained ventricular arrhythmia at electrophysiological testing. On the other hand, 2008 ACC/AHA/HRS guidelines recommend ICD implantation as a Class 1b indication for primary prevention in patients with CHD.

To our knowledge, there are only a few documented cases of VT in a patient with CCTGA (Table 1) [6-9]. An alternative therapy is ablation; Baral et al. reported the successful ablation of monomorphic ventricular tachycardia in a 48-year-old woman with CCTGA, Ebstein's malformation of the tricuspid valve, and incessant VT [6]. On the other hand, atrial fibrillation or supraventricular tachycardia is very rare in the follow-up of patients with CCTGA.

Because of the increasing numbers and survival of patients with CHD, physicians will continue to encounter rhythm problems. ICDs and pacemakers do not solve all these problems, and medical therapies like beta-blockers or amiodarone are sometimes needed. Our patient is one of the oldest patients with CCTGA that has been documented in the literature, and interestingly the patient suffered from multiple arrhythmic problems on follow-up.

## Figures and Tables

**Table 1:**
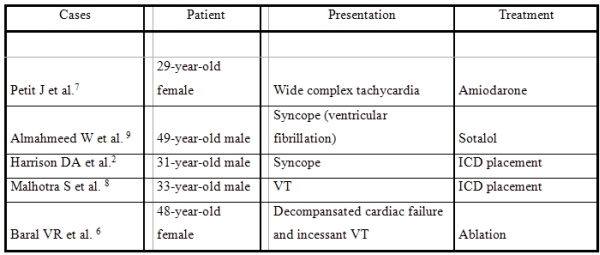
Documented cases of VT in  patients with CCTGA in the literature

VT: ventricular tachycardia, CCTGA: Congenitally corrected transposition of the great arteries, ICD: implantable cardioverter defibrillator, AV: atrioventricular
